# West Nile Virus Lineage 2 Overwintering in Italy

**DOI:** 10.3390/tropicalmed7080160

**Published:** 2022-07-31

**Authors:** Giulia Mencattelli, Federica Iapaolo, Andrea Polci, Maurilia Marcacci, Annapia Di Gennaro, Liana Teodori, Valentina Curini, Valeria Di Lollo, Barbara Secondini, Silvia Scialabba, Marco Gobbi, Elisabetta Manuali, Cesare Cammà, Roberto Rosà, Annapaola Rizzoli, Federica Monaco, Giovanni Savini

**Affiliations:** 1 Istituto Zooprofilattico Sperimentale dell’Abruzzo e del Molise, 64100 Teramo, Italy; f.iapaolo@izs.it (F.I.); a.polci@izs.it (A.P.); m.marcacci@izs.it (M.M.); a.digennaro@izs.it (A.D.G.); l.teodori@izs.it (L.T.); v.curini@izs.it (V.C.); v.dilollo@izs.it (V.D.L.); b.secondini@izs.it (B.S.); s.scialabba@izs.it (S.S.); c.camma@izs.it (C.C.); f.monaco@izs.it (F.M.); g.savini@izs.it (G.S.); 2 Center Agriculture Food Environment, University of Trento, 38098 Trento, Italy; roberto.rosa@unitn.it; 3 Fondazione Edmund Mach, Research and Innovation Centre, San Michele all’Adige, 38098 Trento, Italy; annapaola.rizzoli@fmach.it; 4 Istituto Zooprofilattico Sperimentale dell’Umbria e delle Marche “Togo Rosati”, 06126 Perugia, Italy; m.gobbi@izsum.it (M.G.); e.manuali@izsum.it (E.M.)

**Keywords:** West Nile virus, flavivirus, birds of prey, overwintering, bird-to-bird transmission, rodent-to-bird transmission, hybrid mosquitoes, surveillance

## Abstract

In January 2022, West Nile virus (WNV) lineage 2 (L2) was detected in an adult female goshawk rescued near Perugia in the region of Umbria (Italy). The animal showed neurological symptoms and died 15 days after its recovery in a wildlife rescue center. This was the second case of WNV infection recorded in birds in the Umbria region during the cold season, when mosquitoes, the main WNV vectors, are usually not active. According to the National Surveillance Plan, the Umbria region is included amongst the WNV low-risk areas. The necropsy evidenced generalized pallor of the mucous membranes, mild splenomegaly, and cerebral edema. WNV L2 was detected in the brain, heart, kidney, and spleen homogenate using specific RT-PCR. Subsequently, the extracted viral RNA was sequenced. A Bayesian phylogenetic analysis performed through a maximum-likelihood tree showed that the genome sequence clustered with the Italian strains within the European WNV strains among the central-southern European WNV L2 clade. These results, on the one hand, confirmed that the WNV L2 strains circulating in Italy are genetically stable and, on the other hand, evidenced a continuous WNV circulation in Italy throughout the year. In this report case, a bird-to-bird WNV transmission was suggested to support the virus overwintering. The potential transmission through the oral route in a predatory bird may explain the relatively rapid spread of WNV, as well as other flaviviruses characterized by similar transmission patterns. However, rodent-to-bird transmission or mosquito-to-bird transmission cannot be excluded, and further research is needed to better understand WNV transmission routes during the winter season in Italy.

## 1. Introduction

West Nile virus (WNV) is a mosquito-borne flavivirus, belonging to the family *Flaviviridae*, genus *Flavivirus* [1]. It is part of the Japanese encephalitis serocomplex, which includes other related viruses such as Usutu, Murray Valley encephalitis, Stratford, Alfui, Kunjin, and Saint Louis encephalitis [2]. WNV is transmitted in nature by vector-competent mosquitoes mainly belonging to the *Culex* genus [3]. Its transmission cycle involves several bird species and orders as main-amplifier hosts and humans, equids, and other animals as incidental, dead-end hosts [3,4]. In humans, 80% of cases are generally asymptomatic, while in about 20% of cases, infection causes mild, flu-like symptoms known as West Nile fever (WNF). West Nile neuro-invasive disease (WNND) occurs in less than 1% of cases, reporting WNV meningitis, encephalitis, or poliomyelitis [2,5]. Among birds, several avian species can be infected by WNV, but corvids and raptors appear to be the most susceptible ones, showing neurological symptoms and deaths [5]. Once infected, horses usually do not have symptoms. In 20% of cases, they can develop clinical and neurological forms [6], which can lead to 30–50% deaths in unvaccinated animals [5]. The severity of symptoms has been often correlated to WNV genetic diversity [7]. Up to now, 8 lineages have been recognized. Among them, WNV lineage 1 (WNV L1) and lineage 2 (WNV L2) are by far the most widely spread and the most virulent, capable of causing numerous cases worldwide [2,8,9].

Nowadays, WNV represents a serious public health concern in Europe. In the last decades, WNV has expanded its geographical range. The number of West Nile disease (WND) cases have been increasing in animals and humans, especially in southern, central, and eastern Europe, where many countries have become endemic [10,11].

Italy is one of the European countries most affected by WNV circulation [12]. Since 2002, four years after the first incursion in the Tuscany region [13,14], the Italian Ministry of Health has implemented a veterinary surveillance plan to monitor the viral introduction and circulation of WNV in the whole country. In Italy, the WNV circulation is currently monitored through an annually updated preparedness and response plan, aiming at limiting the risk of WNV transmission to humans either by mosquitoes or by substances of human origin. The current program, modulated on the basis of seasonality and local epidemiology, includes national integrated human, animal (equids and birds), and entomological surveillance (One Health Surveillance). Viral circulation is monitored from April to November by testing vector-competent mosquitoes, resident birds belonging to target species (*Pica pica*, *Corvus corone cornix*, and *Garrulus glandarius*) or sentinel chickens, wild birds found dead, horses showing nervous symptoms, and humans presenting neuro-invasive disease signs. On the basis of WNV occurrence and the eco-climatic characteristics of the territory, Italy is divided in three areas: (1) high-risk areas, where WNV is circulating or has circulated in at least one of the previous five years and where, therefore, episodes of infection have been repeatedly observed; (2) low-risk areas, where WNV has circulated sporadically in the past or has never circulated but whose eco-climatic characteristics are favorable for viral circulation; and (3) minimum-risk areas, where WNV has never circulated and where, given the eco-climatic characteristics of the territory, the probability of its circulation is considered as minimal (National Plan for Prevention, Surveillance, and Response to Arbovirus 2020–2025).

The Umbria region is classified as a low-risk area. WNV circulation has never been reported, at least up to 2019, when the death of a little grebe (*Tachybaptus ruficollis* subsp. *ruficollis*) was associated with WNV L2 infection [15].

In this report, a second clinical case associated with WNV L2 infection observed in a northern goshawk (*Accipiter gentilis*) in Umbria during the winter season is described.

## 2. Materials and Methods

### 2.1. Case Report

**Figure 1:**
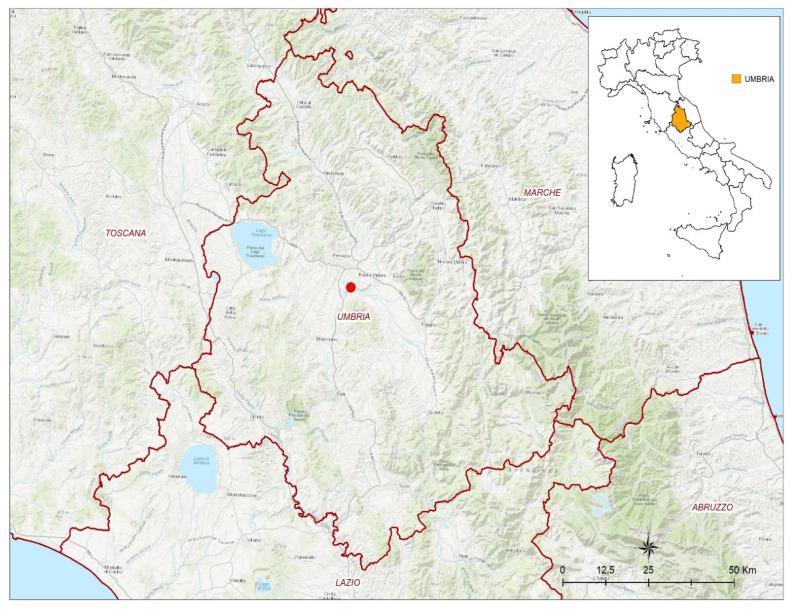
Map of geo-localization site. The northern goshawk was found in the Torgiano municipality (43.0893° N, 12.4410° E) in the Umbria region.

On 4 January 2022, a female adult northern goshawk was rescued in Torgiano, a municipality in the province of Perugia, Italy (Figure 1).

The bird was found on the roof of a private house while trying to defend itself from a mobbing attack by crows. The noise caused by the crows attacking the stunned goshawk attracted the homeowner, who recovered the bird and brought it to a wildlife rescue center (WRC). The northern goshawk showed classical neurological symptoms, including stupor, blepharospasm, and an inability to maintain a normal posture. After an initial improvement of symptoms in which the bird restarted feeding on its own, the clinical signs suddenly worsened, and the bird died on 19 January 2022. The necropsy evidenced a good state of nutrition, generalized pallor of the mucous membranes, and mild splenomegaly. Cerebral edema was also observed. The picture and video of the WNV-infected northern goshawk are reported in Figure S1 and Video S1 (Supplementary Materials).

### 2.2. Laboratory Analyses

#### 2.2.1. Real-Time PCR for WNV and USUV

The brain, heart, kidney, and spleen were collected, pooled, and homogenized in sterile phosphate-buffered saline (PBS). The viral RNA was extracted from 200 μL supernatant using Qiasymphony^®^ DSP automatic instrumentation (Germantown, MD, USA) according to the manufacturer instructions. The extracted RNA was then tested using one-step quantitative reverse transcription polymerase chain reactions (qRT-PCRs) specific for USUV, all known lineages of West Nile virus, and West Nile virus L1 and L2 [16,17,18].

#### 2.2.2. Illumina and Sanger Sequencing

A WNV-positive sample was selected for Illumina and Sanger sequencing. The total RNA was subjected to Turbo DNase treatment (Thermo Fisher Scientific, Waltham, MA, USA) at 37 °C for 20 min and then purified with an RNA Clean and Concentrator-5 Kit (Zymo Research, Irvine, CA, USA). The purified RNA was used for the assessment of the sequence independent single primer amplification (SISPA) protocol [19,20]. In detail, a single-strand cDNA was obtained using reverse transcription (RT) in 20 μL reaction mixture with 5X SSIV buffer, 50 µM random hexamer FR26RV-N 50-GCCGGAGCTCTGCAGATATCNNNNNN-30, 10 mM dNTPs mix, 100 mM DTT, 200 units SuperScript^®^ IV Reverse Transcriptase (Thermo Fisher Scientific, Waltham, MA, USA), and 40 U RNAse OUT RNase inhibitor (Thermo Fisher Scientific, Waltham, MA, USA) following the manufacturer instructions. The reaction was incubated at 23 °C for 10 min, 50 °C for 50 min, and 80 °C for 10 min. To convert the single-stranded cDNA into double-stranded (ds) cDNA, 1 μL (2.5 U) 3′-5′ Klenow Polymerase (New England Biolabs, Ipswich, MA, USA) was directly added to the reaction. The incubation was carried out at 37 °C for 1 h and 75 °C for 10 min. Next, 5 µL of ds cDNA was amplified with a PCR master mix containing 5X Q5 reaction buffer, 10 mM dNTPs, 40 µM random primer FR20 Rv 50 -GCCGGAGCTCTGCAGATATC-30, 0.01 U/µL Q5^®^ High Fidelity DNA polymerase (NEB, New England Biolabs, Ipswich, MA, USA), and 5X Q5 High Enhancer. The reaction was incubated at 98 °C for 10 s, 65 °C for 30 s, 72 °C for 3 min, and 72 °C for 2 min. The PCR product was purified using Expin ^TM^ PCR SV (GeneAll Biotechnology CO., Seoul, Korea) and then quantified using a Qubit^®^ DNA HS Assay Kit (Thermo Fisher Scientific, Waltham, MA, USA). The sample was diluted to obtain a concentration of 100–500 ng, then used for library preparation with an Illumina DNA prep kit, and sequenced with a NextSeq 500 (Illumina Inc., San Diego, CA, USA) using a NextSeq 500/550 Mid Output Reagent Cartridge v2, 300 cycles, and standard 150 bp paired-end reads. After quality control and trimming with Trimmomatic v0.36 (Usadellab, Düsseldorf, Germany) [21] and FastQC tool v0.11.5 (Bioinformatics Group, Babraham Institute, Cambridge, UK) [22,23], reads were *de novo* assembled using SPADES v3.11.1 (Algorithmic Biology Lab, St Petersburg, Russia) [24]. The contigs obtained were analyzed with BLASTn to identify the best match reference. Mapping of the trimmed reads was then performed using the iVar computational tool [25] to obtain a consensus sequence. In order to close some large gaps, the WNV-positive sample was further sequenced using the Sanger method [26]. Briefly, the total RNA was extracted from the collected sample using a High Pure Viral Nucleic Acid Kit (Roche Diagnostics GmbH, Roche Applied Science, 68298 Mannheim, Germany) according to the manufacturer instructions and collected in 45 μL elution buffer prewarmed at 72 °C. The complete WNV-coding DNA sequences (cds) of the polyprotein precursor gene was amplified using 13 WNV primer pairs able to amplify 13 overlapping regions of the genome (the primer sequences are available upon request). Gel-based RT-PCR was performed using a Transcriptor One-Step RT-PCR kit (Roche Diagnostics Deutschland GmbH, Mannheim, Germany) as described by the manufacturer instructions. The RT-PCR cycling conditions for the amplification were 50 °C for 15 min and 94 °C for 7 min, followed by 35 cycles of denaturation at 94 °C for 10 s, annealing at 57.5 °C for 30 s, and extension at 68 °C for 4 min and 30 s, followed by 1 extension cycle performed at 68 °C for 7 min. The gel-based RT-PCR amplicons were purified with a Qiaquick PCR Purification kit (Qiagen, Leipzig, Germany). The purified amplicons and the 13 WNV sequencing primers were sent to an external service, Eurofins Genomics (Eurofins Genomics, Germany GmbH, Anzinger Str. 7a, 85560 Ebersberg, Germany), to perform sequencing in both directions. The obtained sequences were analyzed with SeqScape v3.0 (Thermo Fisher Scientific, Waltham, MA, USA).

#### 2.2.3. Phylogenetic Analysis

A phylogenetic analysis was conducted including 62 WNV L2 genome sequences publicly available. Specifically, 57 complete and 5 partial genome sequences representative of different geographic regions and identified in different hosts were downloaded from Genbank. In addition, three sequences were added as outgroups: WNV L1 Italy 2020 (MW627239), WNV L1 France 2015 (MT863559), and Koutango virus (KOUTV) Senegal 2013 (EU082200). All 66 sequences were aligned using the MAFFT online alignment program (https://mafft.cbrc.jp/alignment/server/, accessed on: 15 April 2022) and curated using BioEdit v. 7.2.5.0 software (https://bioedit.software.informer.com/7.2/, Bioedit Company, Manchester, UK, accessed on: 15 April 2022). The WNV sequence alignment and the metadata of the WNV strains used for the present study are reported in File S1 and Table S1, respectively (Supplementary Materials). Bayesian phylogenetic inference (BI) was performed using a Bayesian Evolutionary Analysis by Sampling Tree (BEAST) software package version 2.6.3 (http://www.beast2.org/, University of Auckland, Auckland 1142, New Zealand) [27,28]. In detail, using the interface program called Bayesian Evolutionary Analysis Utility (BEAUti) included in the BEAST package, the amino acid sequence alignment was uploaded by choosing a gamma-site model with a gamma category count of 4, as well as invariant sites model (GTR + Γ + I) [28]. A family of Bayesian Markov chain Monte Carlo (MCMC) algorithms with 10 independent MCMC runs with up to 100,000,000 generations was used to perform the inference. Using TreeAnnotator v.2.6.3 (https://www.beast2.org/treeannotator/), trees were summarized in a maximum-clade-credibility tree with common ancestor heights after a 10% burnin percentage [28]. Tracer v 1.7.1 (available at http://beast.bio.ed.ac.uk/Tracer, accessed on: 16 April 2022) was used to ensure convergence during the MCMC runs. Finally, the FigTree v2.6.3 program (http://tree.bio.ed.ac.uk/software/) allowed the estimation of a maximum-likelihood tree [29].

## 3. Results

The northern goshawk rescued in the Umbria region in January 2022 was positive for WNV L2 (Ct 25) and turned out negative for WNV L1 and USUV.

The Illumina sequencing run produced a total of 15,763,124 reads. BLASTn analysis was performed to identify the closest publicly available sequence in the GenBank database. The best match (99.49%) was with the West Nile virus isolate of Nea Santa-Greece-2010 (accession no. HQ537483), and this sequence was used to perform mapping with the iVar tool [25]. This analysis produced a consensus sequence with a horizontal coverage (HCov) of 57% and a mean vertical coverage of 575,535, probably due to the low quality of the RNA sample.

The large gaps were partially filled with Sanger sequencing data, and a consensus sequence of 11.056 nt in length was obtained (HCov 91%) and published in the NCBI database under acc. no ON032498 and the NCBI sequence name of 15935/22.

**Figure 2:**
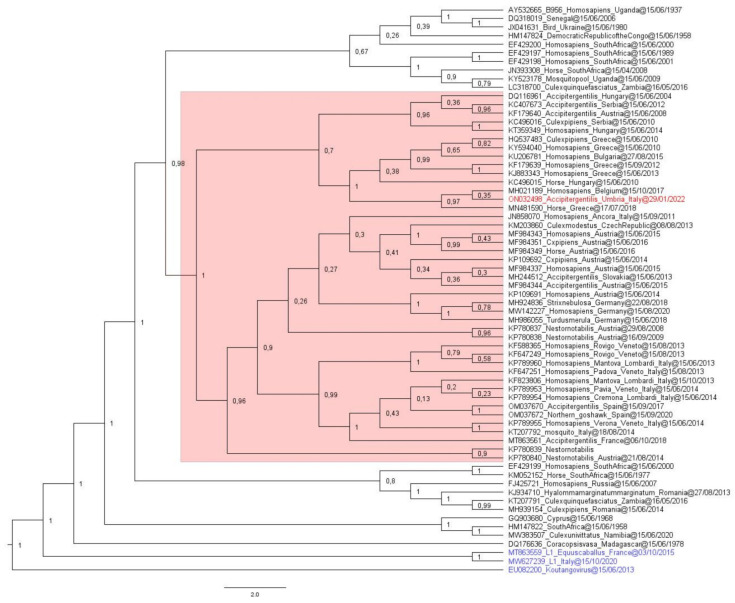
The evolutionary analysis inferred by using the maximum-likelihood method and the general time reversible model is illustrated. The tree with the highest log-likelihood is shown. The percentage of trees in which the associated taxa clustered together is displayed next to the branches. Initial trees for the heuristic search were obtained by applying the neighbor-joining method to a matrix of pairwise distances estimated using the maximum composite likelihood (MCL) approach. A discrete Gamma distribution was used to model evolutionary rate differences among the sites (4 categories). The evolutionary distances were computed using the optimal GTR + Γ + I model, with 2000 Γ-rate categories and 5000 bootstrap replications using the Shimodaira–Hasegawa (SH) test. Ten independent MCMC runs with up to 100 million generations were performed to ensure the convergence of the estimates. GenBank accession numbers are indicated for each strain, with country, lineage, and year of isolation. The genome sequence ON032498 WNV L2 (HCov 91%), obtained from the goshawk organ homogenate, is highlighted in red. The WNV L1 MW627239 (Italy 2020) and MT863559 (France 2015), and Koutango virus (KOUTV) EU082200 (Senegal 2013), chosen as outgroups, are highlighted in blue. The new strain (ON032498) showed high genetic similarity with the central-southern European WNV L2 clade, highlighted in pink, with a posterior probability of 100%.

Phylogenetic analysis placed 15935/22 NCBI in the same cluster as the other central-southern European WNV L2 sequences [30] (highlighted in pink in Figure 2) with a posterior probability of 100%.

## 4. Discussion

This study reported the second evidence of WNV L2 circulation in the Umbria region. The current National Surveillance Plan includes Umbria amongst those areas whose eco-climatic characteristics are favorable for viral circulation but where WNV has never or sporadically circulated in the past (National Plan for Prevention, Surveillance, and Response to Arbovirus 2020–2025). In fact, no WNV circulation has ever been reported in the territory, neither in humans [31] nor in animals, before 2019, when the virus was detected for the first time in a little grebe [15]. Interestingly, in both reported Umbrian cases, WNV was detected in the winter months (December and January), a period when, normally, the most common vector, *Culex pipiens*, is less active.

The northern goshawk (*Accipiter gentilis*) is a medium-sized bird of prey belonging to the family of *Accipitridae*. The family also includes other diurnal raptors, such as eagles, buzzards, and harriers [32]. With regard to WNV infection, northern goshawks have been demonstrated to be highly susceptible. In fact, following WNV infection, severe clinical symptoms have often been described in this species and, in general, in raptors [19,30,33,34]. The clinical signs described in this report were compatible with WNV infection. Moreover, the absence of fractures in the bird skull excluded a possible traumatic origin of the observed neurological disorders.

The first aspect that needs to be clarified is the time of infection: did it really occur in winter? In this regard, the occurrence of clinical signs and the direct detection of WNV in the goshawk organs indicates that this case was related to a recent infection. WNV infections in periods of mosquito inactivity have been described, particularly in raptors [33,35,36]. This group of birds is, in fact, characterized by predatory habits [32] and the transmission of WNV by the predation of infected birds has been frequently observed [33,37]. In this reported case, it is then highly probable that the Umbrian goshawk became infected by eating an infected bird. If, on the one hand this explanation indeed clarifies the way the goshawk obtained the infection, on the other hand, it does not explain how the supposed goshawk prey was still infectious in a period of mosquito inactivity. The persistence of infectious WNV for prolonged periods in the organs of birds and, in particular, of *Passeriformes* has been evidenced by many authors [35,38,39,40,41,42]. Persistent infection has been defined as the detection of a virus in host tissues after viremia has subsided [43]. The persistent, high viral loads in organs of birds and, in particular, in those belonging to prey species might sustain WNV transmission to predators also months after mosquito season. The recent finding of WNV L2 in a little grebe collected in the winter months in Umbria [15] indicates that finding birds with WNV-infected organs in the winter months is not uncommon [33,34,38,41,43]. Thus, the symptomatic goshawk found in Umbria last January can be regarded as a case of bird-to-bird WNV oral transmission.

This transmission route has been considered as one of the possible ways of overwintering for WNV [33,34,38,41,43]. Bearing in mind that common prey of goshawks also include rodents and that, also, in these animals infectious WNV has been detected months after infection [44,45], a possible rodent-to-bird transmission cannot be excluded.

Further attention should be given to the *Culex pipiens* complex because of its vector role in WNV transmission [46,47]. Among this complex, the *Culex pipiens* (rural, mainly ornithophilic) and *Culex molestus* (urban, mainly mammophilic) biotypes can interbreed, giving birth to a hybrid form with intermediate ecological features found in a wide set of environments and acting as a WNV bridge-vector from birds to humans [46,47]. Interestingly, while *Cx. pipiens* are well-known to enter diapause during winter, *Cx. molestus* and *Cx. Pipiens*–*Cx. molestus* hybrids actively feed all year round [46] and might, for this reason, have been responsible for the goshawk WNV infection.

Irrespective of whether the goshawk was infected by hybrid mosquitoes or by the predation of infected animals, WNV has indeed been circulating in the winter months. Therefore, the second important issue to be clarified in this report is where the goshawk was infected. According to the National Surveillance Plan, Umbria is classified as a WNV low-risk area, while the neighboring regions (parts of Tuscany, Marche, and Lazio) are high-risk areas (National Plan for Prevention, Surveillance, and Response to Arbovirus 2020–2025). In most parts of Europe, the northern goshawk is a sedentary species [32,48]. In Italy, it is a rather scarce, localized breeder, mainly present in mature forests of the Apennines and the Alps that are especially rich in large trees and are particularly suitable for nest-building, where its common prey are abundant (e.g., squirrels, wood pigeons, woodpeckers, corvids, and rabbits) [32]. In January, even if it further reduces its movements, it can still keep moving for hundreds of kilometers [32]. Because of that and in view of the fact that Umbria has a very small surface (8.456 Km^2^), it is very difficult to determine whether the goshawk became infected in Umbria or in the neighboring regions. Interestingly, during the WNV season 2021, there was no evidence for the circulation of WNV, not only in Umbria but also in the nearby area of central Italy [49]. The lack of WNV infection cases in high-risk areas (Latium and Tuscany) also in wintertime [15,50], when mosquitoes are less active, might suggest that birds have an important role in WNV overwintering. The potential transmission through the oral route in a predatory bird might explain the relatively rapid spread of WNV and of other similar flaviviruses, such as Tick-borne encephalitis virus and Usutu virus (USUV) [50,51,52]. It has to be said that Umbria is endemic for USUV [53] (National Plan for Prevention, Surveillance, and Response to Arbovirus 2020–2025), which is a mosquito-borne virus that shares the same life cycle and patterns of transmissibility with WNV [4]. If the eco-climatic conditions of Umbria are suitable for maintaining the USUV life cycle, they should also be favorable for WNV.

Based on these features and due to recent WNV positivities detected in the territory, the Umbria region was included among the high-risk areas in 2022 by the WNV National Surveillance Plan (National Plan for Prevention, Surveillance, and Response to Arbovirus 2020–2025).

## 5. Conclusions

In conclusion, our study highlighted the circulation of WNV L2 during the winter in Italy. Even though the reported cases remain rare during the cold season, this report is of fundamental importance because it evidences the potential for human transmission when veterinary active surveillance is suspended. It means that we cannot rely on the early warning system for WNV circulation mainly provided by mosquito and bird surveillance to prevent human infection. Further research is needed to better understand the transmission routes and the role of overwintering birds, rodents, and mosquitoes, as well as WNV infection per *os.* in WNV transmission and epidemiology in Italy. Considering the strong WNV circulation in the Italian territory and the observed changes in the seasonal and regional patterns of WNV, with the virus observed lately in winter times, as well as in WNV low-risk areas, this work also highlighted the strong importance of the passive surveillance of wildlife. This should be coupled with the implementation of a harmonized protocol for necroscopies and biological sample collection, including gastro-intestinal contents, in order to obtain additional data needed to clarify the potential cause of death, especially in winter, when the National Surveillance Plan does not include an active search for the virus.

## Data Availability

Sequence data are available via NCBI. The accession numbers for the sequences used can be found in Table S1, Supplementary Materials.

## Supplementary Materials

The following supporting information can be downloaded at: https://www.mdpi.com/article/10.3390/tropicalmed7080160/s1, Figure S1: Picture of WNV-infected Northern goshawk; Video S1: Video of WNV-infected Northern goshawk; File S1: WNV List of Sequence dataset; Table S1: Metadata of WNV strains used for the present study.

## References

[1] Habarugira G., Suen W.W., Hobson-Peters J., Hall R.A., Bielefeldt-Ohmann H. (2020). West Nile Virus: An Update on Pathobiology, Epidemiology, Diagnostics, Control and “One Health” Implications. Pathogens.

[2] Mencattelli G., Ndione M.H.D., Rosà R., Marini G., Diagne C.T., Diagne M.M., Fall G., Faye O., Diallo M., Faye O. (2022). Epidemiology of West Nile Virus in Africa: An Underestimated Threat. PLoS Negl. Trop. Dis..

[3] Popescu C.P., Florescu S.A., Ruta S.M. (2020). West Nile Virus in Central Europe—Pandora’s Box Is Wide Open!. Travel Med. Infect. Dis..

[4] Mancuso E., Cecere J.G., Iapaolo F., Di Gennaro A., Sacchi M., Savini G., Spina F., Monaco F. (2022). West Nile and Usutu Virus Introduction via Migratory Birds: A Retrospective Analysis in Italy. Viruses.

[5] Byas A.D., Ebel G.D. (2020). Comparative Pathology of West Nile Virus in Humans and Non-Human Animals. Pathogens.

[6] Reisen W.K., Hahn D.C. (2007). Comparison of Immune Responses of Brown-Headed Cowbird and Related Blackbirds to West Nile and Other Mosquito-Borne Encephalitis Viruses. J. Wildl. Dis..

[7] Fall G., Di Paola N., Faye M., Dia M., Freire C.C.d.M., Loucoubar C., Zanotto P.M.d.A., Faye O., Sall A.A. (2017). Biological and Phylogenetic Characteristics of West African Lineages of West Nile Virus. PLoS Negl. Trop Dis..

[8] Bakonyi T., Ivanics É., Erdélyi K., Ursu K., Ferenczi E., Weissenböck H., Nowotny N. (2006). Lineage 1 and 2 Strains of Encephalitic West Nile Virus, Central Europe. Emerg. Infect. Dis..

[9] Beck C., Leparc Goffart I., Franke F., Gonzalez G., Dumarest M., Lowenski S., Blanchard Y., Lucas P., de Lamballerie X., Grard G. (2020). Contrasted Epidemiological Patterns of West Nile Virus Lineages 1 and 2 Infections in France from 2015 to 2019. Pathogens.

[10] West Nile Virus Infection. https://www.ecdc.europa.eu/en/west-nile-virus-infection.

[11] Bakonyi T., Haussig J.M. (2020). West Nile Virus Keeps on Moving up in Europe. Euro Surveill.

[12] Rizzo C., Napoli C., Venturi G., Pupella S., Lombardini L., Calistri P., Monaco F., Cagarelli R., Angelini P., Bellini R. (2016). West Nile Virus Transmission: Results from the Integrated Surveillance System in Italy, 2008 to 2015. Eurosurveillance.

[13] Cantile C., Di Guardo G., Eleni C., Arispici M. (2000). Clinical and Neuropathological Features of West Nile Virus Equine Encephalomyelitis in Italy. Equine Vet. J..

[14] Monaco F., Lelli R., Teodori L., Pinoni C., Di Gennaro A., Polci A., Calistri P., Savini G. (2010). Re-Emergence of West Nile Virus in Italy. Zoonoses Public Health.

[15] Giglia G., Mencattelli G., Lepri E., Agliani G., Gobbi M., Gröne A., van den Brand J.M.A., Savini G., Mandara M.T. (2022). West Nile Virus and Usutu Virus in Wild Birds from Rescue Centers, a Post-Mortem Monitoring Study from Central Italy. bioRxiv.

[16] Del Amo J., Sotelo E., Fernández-Pinero J., Gallardo C., Llorente F., Agüero M., Jiménez-Clavero M.A. (2013). A Novel Quantitative Multiplex Real-Time RT-PCR for the Simultaneous Detection and Differentiation of West Nile Virus Lineages 1 and 2, and of Usutu Virus. J. Virol. Methods.

[17] Vázquez A., Herrero L., Negredo A., Hernández L., Sánchez-Seco M.P., Tenorio A. (2016). Real Time PCR Assay for Detection of All Known Lineages of West Nile Virus. J. Virol. Methods.

[18] Cavrini F., Della Pepa M.E., Gaibani P., Pierro A.M., Rossini G., Landini M.P., Sambri V. (2011). A Rapid and Specific Real-Time RT-PCR Assay to Identify Usutu Virus in Human Plasma, Serum, and Cerebrospinal Fluid. J. Clin. Virol..

[19] Mencattelli G., Iapaolo F., Monaco F., Fusco G., de Martinis C., Portanti O., Di Gennaro A., Curini V., Polci A., Berjaoui S. (2022). West Nile Virus Lineage 1 in Italy: Newly Introduced or a Re-Occurrence of a Previously Circulating Strain?. Viruses.

[20] Marcacci M., De Luca E., Zaccaria G., Di Tommaso M., Mangone I., Aste G., Savini G., Boari A., Lorusso A. (2016). Genome Characterization of Feline Morbillivirus from Italy. J. Virol. Methods.

[21] Bolger A.M., Lohse M., Usadel B. (2014). Trimmomatic: A Flexible Trimmer for Illumina Sequence Data. Bioinformatics.

[22] Cito F., Pasquale A.D., Cammà C., Cito P. (2018). The Italian Information System for the Collection and Analysis of Complete Genome Sequence of Pathogens Isolated from Animal, Food and Environment. Int. J. Infect. Dis..

[23] Aprea G., Scattolini S., D’Angelantonio D., Chiaverini A., Di Lollo V., Olivieri S., Marcacci M., Mangone I., Salucci S., Antoci S. (2020). Whole Genome Sequencing Characterization of HEV3-e and HEV3-f Subtypes among the Wild Boar Population in the Abruzzo Region, Italy: First Report. Microorganisms.

[24] Bankevich A., Nurk S., Antipov D., Gurevich A.A., Dvorkin M., Kulikov A.S., Lesin V.M., Nikolenko S.I., Pham S., Prjibelski A.D. (2012). SPAdes: A New Genome Assembly Algorithm and Its Applications to Single-Cell Sequencing. J. Comput. Biol..

[25] Grubaugh N.D., Gangavarapu K., Quick J., Matteson N.L., De Jesus J.G., Main B.J., Tan A.L., Paul L.M., Brackney D.E., Grewal S. (2019). An Amplicon-Based Sequencing Framework for Accurately Measuring Intrahost Virus Diversity Using PrimalSeq and IVar. Genome Biol..

[26] Verma M., Kulshrestha S., Puri A. (2017). Genome Sequencing. Methods Mol. Biol..

[27] Nei M., Kumar S. (2000). Molecular Evolution and Phylogenetics.

[28] Drummond A.J., Suchard M.A., Xie D., Rambaut A. (2012). Bayesian Phylogenetics with BEAUti and the BEAST 1.7. Mol. Biol. Evol..

[29] Price M.N., Dehal P.S., Arkin A.P. (2010). FastTree 2—Approximately Maximum-Likelihood Trees for Large Alignments. PLoS ONE.

[30] Aguilera-Sepúlveda P., Napp S., Llorente F., Solano-Manrique C., Molina-López R., Obón E., Solé A., Jiménez-Clavero M.Á., Fernández-Pinero J., Busquets N. (2022). West Nile Virus Lineage 2 Spreads Westwards in Europe and Overwinters in North-Eastern Spain (2017–2020). Viruses.

[31] Riccò M., Peruzzi S., Balzarini F. (2021). Epidemiology of West Nile Virus Infections in Humans, Italy, 2012-2020: A Summary of Available Evidences. Trop Med. Infect. Dis..

[32] Peterson R., Mountfort G., Hollom P.A., Pandolfi M., Frugis S. Guida Degli Uccelli d’Europa. Atlante Illustrato a Colori. Libri—Amazon.It. https://www.amazon.it/uccelli-dEuropa-Atlante-illustrato-colori/dp/8874130473.

[33] Vidaña B., Busquets N., Napp S., Pérez-Ramírez E., Jiménez-Clavero M.Á., Johnson N. (2020). The Role of Birds of Prey in West Nile Virus Epidemiology. Vaccines.

[34] Nemeth N.M., Kratz G.E., Bates R., Scherpelz J.A., Bowen R.A., Komar N. (2009). Clinical Evaluation and Outcomes of Naturally Acquired West Nile Virus Infection in Raptors. J. Zoo Wildl. Med..

[35] Garmendia A.E., Van Kruiningen H.J., French R.A., Anderson J.F., Andreadis T.G., Kumar A., West A.B. (2000). Recovery and Identification of West Nile Virus from a Hawk in Winter. J. Clin. Microbiol..

[36] Ip H.S., Van Wettere A.J., McFarlane L., Shearn-Bochsler V., Dickson S.L., Baker J., Hatch G., Cavender K., Long R., Bodenstein B. (2014). West Nile Virus Transmission in Winter: The 2013 Great Salt Lake Bald Eagle and Eared Grebes Mortality Event. PLoS Curr..

[37] Nemeth N., Gould D., Bowen R., Komar N. (2006). Natural and Experimental West Nile Virus Infection in Five Raptor Species. J. Wildl. Dis..

[38] Komar N., Langevin S., Hinten S., Nemeth N., Edwards E., Hettler D., Davis B., Bowen R., Bunning M. (2003). Experimental Infection of North American Birds with the New York 1999 Strain of West Nile Virus. Emerg. Infect. Dis..

[39] Montecino-Latorre D., Barker C.M. (2018). Overwintering of West Nile Virus in a Bird Community with a Communal Crow Roost. Sci. Rep..

[40] Wheeler S.S., Vineyard M.P., Woods L.W., Reisen W.K. (2012). Dynamics of West Nile Virus Persistence in House Sparrows (Passer Domesticus). PLoS Negl. Trop. Dis..

[41] Reisen W.K., Fang Y., Lothrop H.D., Martinez V.M., Wilson J., Oconnor P., Carney R., Cahoon-Young B., Shafii M., Brault A.C. (2006). Overwintering of West Nile Virus in Southern California. J. Med. Entomol..

[42] Conte A., Candeloro L., Ippoliti C., Monaco F., Massis F.D., Bruno R., Sabatino D.D., Danzetta M.L., Benjelloun A., Belkadi B. (2015). Spatio-Temporal Identification of Areas Suitable for West Nile Disease in the Mediterranean Basin and Central Europe. PLoS ONE.

[43] Wheeler S.S., Langevin S.A., Brault A.C., Woods L., Carroll B.D., Reisen W.K. (2012). Detection of Persistent West Nile Virus RNA in Experimentally and Naturally Infected Avian Hosts. Am. J. Trop Med. Hyg..

[44] Tesh R.B., Siirin M., Guzman H., Travassos da Rosa A.P.A., Wu X., Duan T., Lei H., Nunes M.R., Xiao S.-Y. (2005). Persistent West Nile Virus Infection in the Golden Hamster: Studies on Its Mechanism and Possible Implications for Other Flavivirus Infections. J. Infect. Dis..

[45] Appler K.K., Brown A.N., Stewart B.S., Behr M.J., Demarest V.L., Wong S.J., Bernard K.A. (2010). Persistence of West Nile Virus in the Central Nervous System and Periphery of Mice. PLoS ONE.

[46] Vogels C.B.F., van de Peppel L.J.J., van Vliet A.J.H., Westenberg M., Ibañez-Justicia A., Stroo A., Buijs J.A., Visser T.M., Koenraadt C.J.M. (2015). Winter Activity and Aboveground Hybridization Between the Two Biotypes of the West Nile Virus Vector Culex Pipiens. Vector Borne Zoonotic Dis..

[47] Di Luca M., Toma L., Boccolini D., Severini F., La Rosa G., Minelli G., Bongiorno G., Montarsi F., Arnoldi D., Capelli G. (2016). Ecological Distribution and CQ11 Genetic Structure of Culex Pipiens Complex (Diptera: Culicidae) in Italy. PLoS ONE.

[48] Brown L., Amadon D. (1989). Eagles, Hawks and Falcons of the World.

[49] Bollettino_WND_2021_19 (1).Pdf—Adobe Cloud Storage. https://acrobat.adobe.com/link/file/?x_api_client_id=chrome_extension_viewer&uri=urn%3Aaaid%3Asc%3AUS%3A9868069d-ee54-4d0a-9351-4da5a42ffa6b&filetype=application%2Fpdf&size=1302218.

[50] Bollettino_WND_2022_3.Pdf—Adobe Cloud Storage. https://acrobat.adobe.com/link/file/?x_api_client_id=chrome_extension_viewer&uri=urn%3Aaaid%3Asc%3AUS%3A359a79f6-a813-4fdd-90b8-b52904f3ca1b&filetype=application%2Fpdf&size=1468985.

[51] Riccò M. (2021). Epidemiology of Tick-Borne Encephalitis in North-Eastern Italy (2017–2020): International Insights from National Notification Reports. Acta Biomed..

[52] Toscana_2020.Pdf—Adobe Cloud Storage. https://acrobat.adobe.com/link/file/?x_api_client_id=chrome_extension_viewer&uri=urn%3Aaaid%3Asc%3AUS%3A35ca9c99-25e9-406b-b502-28c485af700&filetype=application%2Fpdf&size=344881.

[53] (2017). Mancini Mosquito Species Involved in the Circulation of West Nile and Usutu Viruses in Italy. Vet. Ital..

