# Supplementary material for: West Nile Virus Lineage 2 Overwintering in Italy
Source: Trop Med Infect Dis. 2022 Jul 31;7(8):160. doi: 10.3390/tropicalmed7080160 (PMC9414329; doi:10.3390/tropicalmed7080160)
Supplement: Supplementary file 1 [file tropicalmed-07-00160-s001.zip › List of Sequence Dataset _File S1.pdf]

Table S1. Metadata of WNV strains used for the present study

Strain number	Viral Species	Isolation Material	Host	Country	Year of isolation	Accession Number	Genome
2022.TE.15935	WNV L2	Homogenate	Northern goshawk	Italy	2022	ON032498	91% genome
HS101_08	WNV L2	Brain	Horse	South Africa	2008	JN393308	Complete genome
Italy/2011/AN-2	WNV L2	Urine	Human	Italy	2011	JN858070	Complete genome
LEIV-3266Ukr	WNV L2	-	Bird	Ukraine	1980	JX041631	Complete genome
Sad/12	WNV L2	-	Northern goshawk	Serbia	2012	KC407673	Complete genome
578/10	WNV L2	-	Horse	Hungary	2010	KC496015	Complete genome
Nea Santa-Greece-2010	WNV L2	Mosquito pool	*Culex pipiens*	Greece	2010	HQ537483	Complete genome
Greece/2012/Kavala/39.1	WNV L2	Urine	Human	Greece	2012	KF179639	Complete genome
Austria/2008_gh	WNV L2	Vero P2	Goshawk	Austria	2008	KF179640	Complete genome
Italy/2013/Rovigo/32.1	WNV L2	Urine	Human	Italy	2013	KF588365	Complete genome
Italy/2013/Rovigo/33.2	WNV L2	Urine	Human	Italy	2013	KF647249	Complete genome

goshawk-Hungary/04	WNV L2	-	Goshawk	Hungary	2004	DQ116961	Complete genome
ArD76104	WNV L2	SMB P3, C6/36 P1	Mosquito	Senegal	2006	DQ318019	Complete genome
SA381/00	WNV L2	-	Human	South Africa	2000	EF429199	Complete genome
H442	WNV L2	-	Human	South Africa	1958	EF429200	Complete genome
Italy/2013/Padova/34.1	WNV L2	Urine	Human	Italy	2013	KF647251	Complete genome
Italy/2013/Mantova/40.1	WNV L2	Urine	Human	Italy	2013	KF823806	Partial genome
Greece/2013/Xanthi_1	WNV L2	Urine	Human	Greece	2013	KJ883343	Complete genome
349/77	WNV L2	-	Horse	South Africa	1977	KM052152	Partial genome
Cz 13-104	WNV L2	Mosquito pool	*Culex modestus*	Czech Republic	2013	KM203860	Complete genome
blood donor/Vienna/2014	WNV L2	Blood donor serum	Human	Austria	2014	KP109691	Complete genome
Cx. pipiens/Vienna/2014	WNV L2	Mosquito pool	*Culex pipiens*	Austria	2014	KP109692	Complete genome
Sepperl	WNV L2	-	Kea	Austria	2008	KP780837	Complete genome

Mismo	WNV L2	-	Kea	Austria	2009	KP780838	Complete genome
Berliner	WNV L2	-	Kea	Austria	2011	KP780839	Complete genome
Tammy	WNV L2	-	Kea	Austria	2014	KP780840	Complete genome
Italy/2014/Pavia1	WNV L2	-	Human	Italy	2014	KP789953	Complete genome
Italy/2014/Cremona2	WNV L2	-	Human	Italy	2014	KP789954	Complete genome
Italy/2014/Verona/35.1	WNV L2	Urine	Human	Italy	2014	KP789955	Complete genome
Italy/2013/Mantova/36.1	WNV L2	Urine	Human	Italy	2013	KP789960	Complete genome
1270/14	WNV L2	-	Mosquito	Italy	2014	KT207792	Complete genome
2014/hun	WNV L2	Urine	Human	Hungary	2014	KT359349	Complete genome
Bulgaria/2015/Sofia	WNV L2	-	Human	Bulgaria	2015	KU206781	Complete genome
341/2010	WNV L2	Blood	Human	Greece	2010	KY594040	Complete genome
Zmq16m11	WNV L2	-	*Culex quinquefasciatus*	Zambia	2016	LC318700	Complete genome

Blood donor 1/Austria/2015	WNV L2	-	Human	Austria	2015	MF984337	Complete genome
Patient 2/Austria/2015	WNV L2	Urine	Human	Austria	2015	MF984343	Complete genome
Goshawk/Austria/2015	WNV L2	Brain	Goshawk	Austria	2015	MF984344	Complete genome
Horse 1/Austria/2016	WNV L2	Brain	Horse	Austria	2016	MF984349	Complete genome
Cx. pipiens 1/Austria/2016	WNV L2	Mosquito pool	*Culex pipiens*	Austria	2016	MF984351	Complete genome
WNV/Belgium/2017/Antwerpen	WNV L2	-	Human	Belgium	2017	MH021189	Complete genome
286.B/2013/Velky Biel/SVK	WNV L2	Brain	Northern goshawk	Slovakia	2013	MH244512	Complete genome
ED-I-33/18-UM	WNV L2	Spleen	Great grey owl	Germany	2018	MH924836	Complete genome
1382/2018/Berlin/Ger	WNV L2	-	Common blackbird	Germany	2018	MH986055	Complete genome
Novi Sad-2010	WNV L2	-	*Culex pipiens*	Serbia	2010	KC496016	Complete genome
Reb_VLG_07_H	WNV L2	Brain	Human	Russia	2007	FJ425721	Partial genome
B956	WNV L2	TX suckling mouse P2; C636 cells P1	Human	Uganda	1937	AY532665	Complete genome

West Nile virus SPU116/89	WNV L2	-	Human	South Africa	1989	EF429197	Complete genome
SA93/01	WNV L2	-	Human	South Africa	2001	EF429198	Complete genome
UG2274/Uganda/2009	WNV L2	Mosquito pool	*Culex neavei*	Uganda	2009	KY523178	Complete genome
Madagascar-AnMg798	WNV L2	-	Greater vasa parrot	Madagascar	1978	DQ176636	Complete genome
-	WNV L2	-	-	Democratic Republic of the Congo	1958	HM147824	Complete genome
Hyalomma/Romania/2013	WNV L2	-	*Hyalomma marginatum marginatum*	Romania	2013	KJ934710	Partial genome
792/14	WNV L2	-	Mosquito	Italy	2014	KT207791	Complete genome
LE6/2020/Leipzig/Germany	WNV L2	-	Human	Germany	2020	MW142227	Complete genome
DD84c	WNV L2	-	*Culex pipiens s.l.*	Romania	2014	MH939154	Complete genome
7025/France/2018	WNV L2	-	Northern goshawk	France	2018	MT863561	Complete genome
-	WNV L2	-	-	South Africa	1958	HM147822	Complete genome

M6646-NA-2020	WNV L2	-	*Culex univittatus*	Namibia	2020	MW383507	Complete genome
Spain/2020/Northern_goshawk/AC923	WNV L2	-	Northern goshawk	Spain	2020	OM037672	Complete genome
Q3574-5	WNV L2	-	-	Cyprus	1968	GQ903680	Partial genome
S14c_GrM_2018/Thessaloniki	WNV L2	-	Horse	Greece	2018	MN481590	Complete genome
Spain/2017/Northern_goshawk/AC568	WNV L2	-	Northern goshawk	Spain	2017	OM037670	Complete genome
Akela/France/2015	WNV L1	-	Horse	France	2015	MT863559	Complete genome
TE_362447_2020	WNV L1	-	Northern goshawk	Italy	2020	MW627239	Partial genome
Dak Ar D 5443	KOUTV	-	Mosquito	Senegal	2013	EU082200	Partial genome
