# Supplementary figures and images for: West Nile Virus Lineage 2 Overwintering in Italy
Source: Trop Med Infect Dis. 2022 Jul 31;7(8):160. doi: 10.3390/tropicalmed7080160 (PMC9414329; doi:10.3390/tropicalmed7080160)

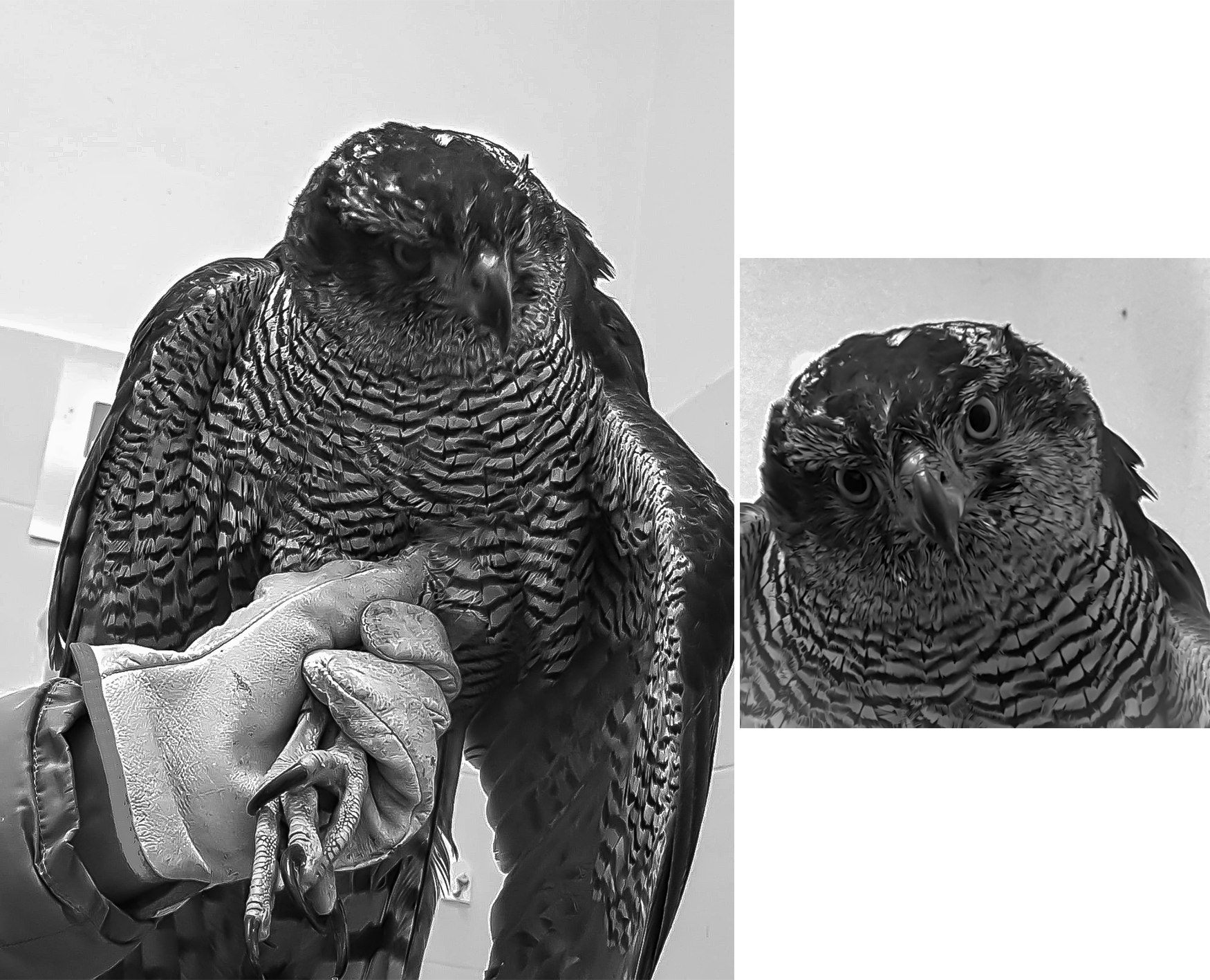

Supplement: Supplementary file 1 [file tropicalmed-07-00160-s001.zip › WNV infected Northern goshawk_Figure S1.png]
